# Group CBT targeting hostile attribution bias in adolescents and young adults with ASD traits

**DOI:** 10.3389/fpsyt.2026.1796850

**Published:** 2026-04-22

**Authors:** Hidehiro Umehara, Tomoya Takeda, Kanae Matsuura, Koushi Irizawa, Yasuko Abe, Tarishi Masuda, Yuichiro Kamiyama, Naoki Yamada, Shusuke Numata

**Affiliations:** 1 Accessibility Division, Health Service, Counseling and Accessibility Center, Tokushima University, Tokushima, Japan; 2 Department of Psychiatry, Tokushima University Hospital, Tokushima, Japan; 3 Department of Psychological Sciences, University of Human Environments, Ehime, Japan; 4 Medical Affairs Division, Tokushima University Hospital, Tokushima, Japan; 5 Department of Psychiatry, Graduate School of Biomedical Sciences, Tokushima University, Tokushima, Japan

**Keywords:** adolescence, autism spectrum disorder, group cognitive behavioral therapy, hostile attribution bias, quality of life

## Abstract

**Background:**

Adolescence is characterized by heightened self-consciousness and sensitivity to social evaluations. During this period, hostile attribution bias—interpreting ambiguous social cues as hostile—can lower quality of life (QOL) and contribute to future mental health problems. Adolescents with autism spectrum disorder (ASD) show similar difficulties, often more pronounced due to their cognitive style and interpersonal vulnerabilities. Group cognitive behavioral therapy (CBT) aims to correct such biases through structured cognitive and social experiences. This study evaluated the psychological effects of group CBT on hostile attribution bias, social functioning, and QOL in adolescents and young adults with ASD traits.

**Methods:**

We conducted an 8-session group CBT program focusing on hostile attribution bias and suspiciousness in 21 adolescents and young adults with ASD traits attending a hospital psychiatric outpatient department. The 15 participants who completed the program were included in analyzes. Psychological indices included hostile attribution bias (Ambiguous Intentions Hostility Questionnaire), social functioning (Social Responsiveness Scale, Second Edition [SRS-2]), and subjective QOL. Pre–post changes were quantified as change rates ((post − pre)/pre × 100). Group-level changes were tested with paired analyzes; exploratory associations among change rates were examined using Spearman correlations.

**Results:**

Group CBT significantly improved hostile attribution bias (effect size [ES] = 0.698, p = 0.017), social communication and interaction (SRS-2; ES = 0.780, p = 0.012), and subjective QOL (ES = 0.752, p = 0.011). Exploratory individual-level analyzes showed a discordant pattern: smaller reductions in hostile attribution bias (less negative change rates) were associated with greater increases in subjective QOL (ρ = 0.597, p = 0.019).

**Conclusions:**

This pilot study suggests that group CBT may reduce hostile attribution bias and improve QOL and social functioning in adolescents and young adults with ASD traits. Notably, the positive correlation between hostile attribution bias change rates and QOL change rates suggests that greater QOL gains were not necessarily accompanied by larger reductions in hostile attribution bias, indicating that improvements in cognitive bias and perceived well-being may arise through partly distinct or non-linear pathways rather than a simple one-to-one relationship.

**Clinical trial registry:**

University Hospital Medical Information Network (UMIN000030140).

## 1 Introduction

Adolescence is a developmental stage characterized by rapid growth in interpersonal relationships and self-awareness along with a simultaneous increase in psychological vulnerability. Adolescents are particularly prone to heightened sensitivity toward ambiguous social cues. During this developmental stage, ambiguous interpersonal signals, such as peer tones of voice or facial expressions, are more readily perceived as socially threatening. Increased social threat sensitivity has been identified as a critical risk factor for internalizing problems including heightened anxiety and emotional maladjustment (1). This cognitive tendency to misinterpret the ambiguous behaviors of others as hostile is termed hostile attribution bias (2), which can contribute to interpersonal misunderstandings and aggressive responses. This bias has been linked to various psychological difficulties including depression, anxiety, social withdrawal, and diminished subjective quality of life (QOL) (3). Although developmental trajectories of hostile attribution bias have been theoretically proposed, they have not been firmly established through empirical research. Studies conducted in Japan have reported persistently high levels of hostile attribution bias among university students (4).

Cross-cultural findings further suggest that the underlying structure of hostile attribution biases broadly consistent across societies; however, its intensity and relational patterns vary according to sociocultural norms. For example, young adults in interdependent cultures such as Japan tend to show lower hostile attributions toward close in-group members, whereas Western samples often display higher levels of hostile interpretations across a wider range of social contexts (5). Despite these cultural variations, HAB is consistently associated with lower subjective well-being and greater emotional distress across countries, indicating that negatively interpreting ambiguous social cues has universally detrimental effects on mental health (6). Therefore, reducing hostile attribution tendencies is an important target for mental health interventions regardless of cultural background.

Adolescence is a critical period for the formation and modification of hostile attribution bias, which develops through interactions among social sensitivity, emotional regulation, past interpersonal experiences, and personality traits. Elevated hostile attribution bias may also perpetuate itself by eliciting further peer rejection, thus reinforcing a vicious cycle (7). Without appropriate environmental support this bias may increase over time (2). Notably, developmental research emphasizes that early hostile attributions are influential precursors to later social cognition and behavior, thus highlights adolescence as a pivotal phase in the formation and modification of social-cognitive biases (8).

As adolescence is a period in which sensitivity to social evaluation and immature theory of mind, adolescents with autism spectrum disorder (ASD) are more prone to heightened suspiciousness and hostile attribution bias (9–13). However, psychological interventions for ASD have mainly focused on psychoeducation or social skills training with limited attention given to suspiciousness or attributional biases (14).

Cognitive behavioral therapy (CBT) is regarded as an effective and safe intervention for psychological problems in adolescents, with efficacy comparable to or greater than that of pharmacotherapy (7, 8) CBT is a method that reconstructs distorted cognitions underlying emotions and behaviors; correcting misattributions and hostile attribution bias may reduce aggressive responses, thereby contributing to the mitigation of aggression and QOL improvement in adolescents (2, 3).

One of the most widely implemented psychosocial interventions worldwide for psychiatric disorders, particularly schizophrenia, is Social Cognition and Interaction Training (SCIT), a group CBT that addresses multiple cognitive biases underlying delusional thinking, including suspiciousness and hostile attribution bias (15). We previously adapted the SCIT for adolescents in middle and high school by simplifying its contents to ensure accessibility and demonstrated its feasibility and potential to improve cognitive biases and subjective QOL in adolescents with ASD (14). CBT can be delivered in individual or group formats; the choice is influenced by age, psychological characteristics, and treatment context. According to a systematic review and meta-analysis by Guo et al. (14), the efficacy of individual and group CBT is not significantly different, although in adolescence, individual CBT may demonstrate greater efficacy (standardized mean difference = −0.77, 95% confidence interval: −1.51 to −0.02). However, group CBT offers unique advantages not found in individual formats, such as opportunities for normalization, positive peer modeling, reinforcement, social support, exposure to social situations, and greater cost-effectiveness (16).

In the present study, we implemented a group CBT program that focused on hostile attribution bias and suspiciousness among adolescents with pronounced distortions of social cognition. We primarily evaluated psychological indices (bias tendencies and QOL) before and after group CBT. In addition, as an exploratory ancillary component, we collected salivary samples to examine endogenous oxytocin reactivity. Because salivary oxytocin assessed by a non-extracted immunoassay has limited independent validation and should not be used to support mechanistic claims, these biomarker findings are reported only in the **Supplementary Material** and interpreted cautiously as descriptive information that may inform future work.

## 2 Materials and methods

### 2.1 Participants and intervention

A total of 21 adolescent and young adult outpatients with autism spectrum disorder (ASD) traits, recruited from the Department of Psychiatry, Tokushima University Hospital, participated in this study (**Table 1**). In this study, the term adolescents and young adults was used in a broad sense to include participants aged up to 24 years. Although some participants were older than the conventional age range of adolescents, all were still students without prior work experience, thus considered to be in a moratorium period characterized by continued psychosocial development and dependency.

**Table 1:** Participants’ age, sex, attendance, comorbidities, and oral medications.

No	Age group	Sex	Attendance	Comorbidity category	Medication class
1	12–18	Male	1	Sleep–wake disorders	Sleep-related medication
2	12–18	Female	7	Neurodevelopmental disorders	Stimulant
3	12–18	Male	1	Neurodevelopmental disorders	Stimulant
4	19–24	Female	6	Bipolar and related disorders	Mood stabilizer
5	19–24	Female	1	No comorbid psychiatric disorder	-
6	12–18	Female	8	No comorbid psychiatric disorder	-
7	12–18	Male	6	Neurodevelopmental disorders	Stimulant/Antipsychotic
8	12–18	Female	6	Anxiety disorders	Antidepressant
9	12–18	Female	8	No comorbid psychiatric disorder	Antipsychotic/Anxiolytic
10	19–24	Male	7	Obsessive-compulsive and related disorders	Antidepressant/Antipsychotic
11	12–18	Male	7	Tic disorders	-
12	19–24	Female	7	Anxiety disorders	Antidepressant/Anxiolytic
13	12–18	Female	7	Anxiety disorders	-
14	12–18	Male	8	Trauma- and stressor-related disorders	-
15	12–18	Male	8	Trauma- and stressor-related disorders	-
16	12–18	Male	1	Neurodevelopmental disorders; Disruptive, impulse-control, and conduct disorders	-
17	12–18	Female	8	Neurodevelopmental disorders	-
18	12–18	Female	1	No comorbid psychiatric disorder	-
19	12–18	Female	7	Anxiety disorders	-
20	12–18	Male	6	Depressive disorders	Antidepressant/Sleep-related medication
21	12–18	Male	1	No comorbid psychiatric disorder	-

Attendance indicates the number of group CBT sessions attended (maximum = 8).

To minimize the risk of participant identification, age is reported in ranges, comorbidities are presented using broad DSM-5 diagnostic categories, and medications are summarized by class without specific drug names or dosages.

Two child and adolescent psychiatrists used the Diagnostic and Statistical Manual of Mental Disorders, Fifth Edition (DSM-5) for diagnoses. Participant comorbidities included attention deficit/hyperactivity, sleep-wake, bipolar II, obsessive-compulsive, generalized anxiety, social anxiety, conduct, adjustment, and major depressive disorders. Participants had diverse educational situations, including regular classes, special support classes, correspondence schools, free schools, and school non-attendance. All participants had a total IQ above 80, and none met criteria for intellectual disability. All were considered to have sufficient language comprehension for participation in the present group-based CBT program, although many had difficulties in social communication and required structured support in group settings. No participant presented with severe self-injurious, aggressive, or markedly disruptive behavior that would make participation in a small-group psychotherapy program infeasible. None had previously received psychotherapy. Of all the participants, 10 were taking medication. Fifteen participants who consistently attended the CBT program were included in the final analyzes. Six participants only attended the first session and did not return for the subsequent sessions.

The intervention program was adapted from a previously reported CBT protocol for children and adolescents with ASD (14) with a specific focus on hostile attribution bias and suspiciousness. The program was reorganized from 16 to 8 sessions in response to participant feedback that indicated a 16-session program was too prolonged, and to more specifically focus the intervention on suspiciousness and hostile attribution bias as the primary therapeutic targets; the details are presented in **Table 2**. All sessions were facilitated by the same lead facilitator throughout the program. The lead facilitator was a psychiatrist board-certified in child and adolescent psychiatry and had completed formal training to become a facilitator of the original SCIT program. To enhance treatment fidelity, the content and process of each session were checked against the SCIT Fidelity Scale, which was used to confirm whether the session had maintained content equivalent to the original SCIT framework while implementing the adapted program for this study.

**Table 2:** Content and symptom targets of the eight-session group cognitive behavioral therapy program.

Session	Title	Target concept	Summary of content	Relevance to hostile attribution bias
Session 1	Becoming Aware of Emotional and Cognitive Biases	Relationship between emotions and cognition; introduction to cognitive biases	Participants learn to differentiate between emotions, thoughts, and behaviors using daily-life examples. Introduction to cognitive biases.	Introduces the concept of cognitive biases and overinterpretation of others’ intentions, which are central to hostile attribution bias.
Session 2	Distinguishing Facts from Assumptions	Confidence in one’s own thoughts; differentiation between facts and assumptions	Training to distinguish between facts and assumptions, assess confidence levels, and reflect on thought validity through video-based exercises.	Encourages participants to critically assess the evidence for their hostile interpretations and question cognitive certainty.
Session 3	Recognizing Emotions	Awareness of emotions and primary emotional triggers	Understanding the variety of emotions and their physiological correlates; identifying primary emotions underlying anger through interactive activities.	Explores emotional antecedents (anger) that influence hostile interpretations and promotes awareness of underlying emotions.
Session 4	Dealing with Emotions through Mindfulness	Emotion regulation through mindfulness and relaxation	Introduction to mindfulness and relaxation techniques (abdominal breathing, progressive muscle relaxation) to manage intense emotions.	Presents mindfulness as a strategy to reduce emotional reactivity and mitigate the impact of HAB on interpersonal perception.
Session 5	Understanding Attribution Bias	Causal attribution patterns (self-blame, other-blame, situational)	Participants explore attributional styles and reflect on the emotional and behavioral consequences of self-, other-, and neutral attributions.	Examines how causal explanations of others’ behavior may reinforce or reduce tendencies toward hostile interpretation.
Session 6	Reviewing How We Gather Information	Cognitive biases in information gathering (confirmation bias, filtering)	Illustration of biased information processing, including confirmation bias, jumping to conclusions, and filtering, via experiential games.	Demonstrates how selective information gathering may exacerbate HAB; encourages broader evidence consideration.
Session 7	Recognizing Social Cues	Nonverbal social cues and emotional interpretation	Training in decoding nonverbal emotional cues through facial expression interpretation and multimodal emotion recognition tasks.	Enhances accuracy in reading others’ emotional cues, reducing misinterpretation and biased hostile assumptions.
Session 8	Expressing Thoughts and Listening to Others	Metacognitive reflection and tolerance of diverse perspectives	Participants apply learned skills in perspective-taking and reflective judgment through discussion-based decision-making exercises.	Encourages cognitive flexibility and empathy in social interactions, promoting alternatives to hostile interpretations.

Each group consisted of three to four adolescents and young adults who were unfamiliar with each other prior to the program. The participants, group leaders, and co-leaders remained consistent throughout the sessions. The sessions were conducted once a week on Thursdays from 16:30 to 17:30 (1 hour).

### 2.2 Psychological measures

Three types of psychological measures were obtained: hostile attribution bias using the Ambiguous Intentions Hostility Questionnaire [AIHQ], objective social functioning using the (Social Responsiveness Scale, Second Edition [SRS-2]), and subjective QOL scales (for junior high and high school students).

The AIHQ is a self-report instrument designed to assess hostile attribution bias in ambiguous social situations (17). It presents multiple scenarios depicting everyday interpersonal events (being bumped into, not being invited) categorized as accidental, ambiguous, or intentional. Following each vignette, the participants responded to three self-report anchor questions concerning the perceived intent of the actor, the degree to which the actor deserves blame, and the participant’s anger. In addition, they provided two open-ended responses regarding their interpretation of the actor’s motives and their anticipated behavioral reactions. Responses were scored as follows: one self-reported blame score per vignette and two rater-coded indices derived from the open-ended responses (hostile attribution bias and aggressive response bias). The two open-ended AIHQ responses were coded using a Japanese scoring manual together with an example-based coding guide. For the attribution-of-motive response, hostile attribution bias was rated on a 5-point scale reflecting the extent to which the respondent inferred intentional and harmful hostile motives in the actor’s behavior. For the anticipated behavioral response, aggressive response bias was rated on a 5-point scale reflecting the degree of aggressive retaliation, from non-aggressive/passive responses to overt physical aggression. In parallel, participants provided self-report ratings of intentionality, anger, and blame for each vignette; the mean of these ratings was used as the blame score. Consistent with the scoring manual, scores were averaged separately across ambiguous, intentional, and accidental scenarios.

The SRS-2 is a standardized caregiver-report questionnaire that evaluates social functioning and ASD traits (18). It consists of 65 items rated on a 4-point Likert scale (0–3), covering domains such as social awareness, social motivation, social communication, and restricted interests/repetitive behaviors. Higher scores indicate greater impairment in reciprocal social behavior. In the present study, we employed two subscales, Social Communication and Interaction (SCI) and Restricted and Repetitive Behavior (RRB), rather than the total score, based on their established clinical significance and structural independence. This approach was adopted because impairments in social skills and behavioral flexibility may have distinct relationships with hostile attribution bias and subjective QOL. Moreover, this two-factor structure is consistent with DSM-5 diagnostic criteria for ASD. Although the SRS-2 is standardized for individuals aged 4–18 years, we also administered it to the participants older than 18 years because the Japanese version for adults has not yet been released and all the participants aged 19 years and older were still students without prior work experience. Among the 15 participants who completed the sessions, SRS-2 caregiver-report data could not be obtained from one participant because the caregiver declined to complete the questionnaire.

For subjective QOL assessment, younger individuals require different questionnaire items to assess the same concept of subjective QOL depending on their developmental stage; therefore, two separate scales were used: one for elementary/junior high school students (19) and one for high school students and older (20). Both scales were developed by the same research group (19, 20) and share a common conceptual basis for comprehensively assessing subjective satisfaction across domains such as anxiety, self-esteem, interpersonal relationships, family, school, and health status. Although the raw scores from the two scales are not directly comparable, we standardized them (Z-scores) to statistically adjust for scale differences, allowing integrated analyzes across age groups.

As an exploratory ancillary component, salivary samples were collected to examine endogenous oxytocin reactivity. Because salivary oxytocin assessed by a non-extracted immunoassay has limited independent validation and should not be used to support mechanistic claims, detailed procedures and all oxytocin-related results are reported only in the **Supplementary Material** and are interpreted cautiously as descriptive information.

### 2.3 Statistical analyzes

The primary analyzes evaluated pre–post changes in psychological outcomes following the group CBT program. Normality was tested using the Shapiro–Wilk test. When normality was confirmed, paired *t*-tests were used; otherwise Wilcoxon signed-rank tests were conducted. The subjective QOL scores for the junior high school students and those for the high school students and older were standardized (Z-scores) and integrated.

For the correlation analyzes, rates of change for all psychological scale scores were calculated using the formula (post − pre)/pre × 100; Spearman’s rank correlation coefficients were computed. For each measure, we first computed individual change indices, then standardized these change scores (Z-scores). Because two QOL instruments were used across age groups, QOL change was Z-standardized within each instrument and then pooled.

All statistical analyzes were performed using jamovi (Version 2.6.44; The jamovi project, 2025). Retrieved from https://www.jamovi.org.

### 2.4 Ethical considerations

Ethical approval was obtained for the study from the Ethics Committee of Tokushima University Hospital and the trial was registered with the University Hospital Medical Information Network (UMIN000030140). Written consent was obtained from all participants and their caregivers. When participant consent was obtained, they were informed that they could withdraw from the study at any time and that any interruption would not usually affect their treatment.

## 3 Results

### 3.1 Changes in psychological and physiological indices before and after CBT

The normality tests showed that hostile attribution bias scores, aggressive response bias scores from the AIHQ, and SCI subscale scores from the SRS-2 followed a normal distribution (Shapiro–Wilk test; *p* > 0.05), and paired *t*-tests were conducted. The blame scores from the AIHQ, RRB subscale scores from the SRS-2, and subjective QOL scores did not follow a normal distribution (Shapiro–Wilk test; *p* <.05); therefore, Wilcoxon signed-rank tests were used.

The psychological indices results before and after CBT are presented in **Table 3**. Hostile attribution bias as assessed by the AIHQ showed a significant decrease, indicating that group CBT reduced distortions in social cognition (effect size [ES] = -0.698, *p* = 0.017). Objective social functioning, assessed using the SRS-2 SCI subscale, also significantly decreased, suggesting improvements in objectively rated social functioning from group CBT (ES = -0.780, *p* = 0.012). In addition, the subjective QOL scores increased significantly, indicating enhanced subjective life satisfaction among the participants (ES = 0.752, *p* = 0.011).

**Table 3:** Outcome between pre- and post- CBT.

Variable	pre-CBT		post-CBT			
	mean(SD)	median(Q1-Q3)	mean(SD)	median(Q1-Q3)	*p*	ES
N	15					
Age	16.27 (3.49)					
Sex (%) Male	9 (60.0)					
Female	6 (40.0)					
AIHQ_Hostile attribution bias	5.79 (0.90)		5.24 (0.90)		0.017	-0.698
AIHQ_Aggressive response bias	4.64 (0.76)		4.43 (1.26)		0.465	-0.194
AIHQ_Blame score		7.47 [6.07, 8.83]		7.20 [6.27, 8.27]	0.599	-0.167
SRS-2_SCI	68.71 (10.67)		62.57 (8.37)		0.012	-0.780
SRS-2_RRB		56.00 [53.00, 72.50]		65.00 [56.00, 71.00]	0.893	0.061
subjective QOL_zscore		-0.62 [-0.96, 0.38]		0.00 [-0.62, 0.87]	0.011	0.752

AIHQ, Ambiguous Intentions Hostility Questionnaire; SRS-2_SCI, Social Responsiveness Scale, Second Edition – Social Communication and Interaction subscale; SRS-2_RRB, Social Responsiveness Scale, Second Edition – Restricted and Repetitive Behavior subscale; QOL, Quality of Life; CBT, cognitive behavioral therapy. Oxytocin change rates were derived as (post-session value − pre-session value)/pre-session value. ‘Pre-CBT’ denotes the change rate obtained in Session 1, whereas ‘Post-CBT’ denotes the change rate obtained in Session 8.” For variables with normal distribution, paired t-tests were used to calculate *p*-values, with results reported as means ± standard deviations and effect sizes (ES) expressed as Cohen’s d. For variables without normal distribution, Wilcoxon signed-rank tests were used, with results reported as medians and interquartile ranges (Q1–Q3), and ES expressed as rank-biserial correlations (r).

Exploratory subgroup analyzes were conducted to examine the potential influence of concurrent psychotropic medication use on the main outcomes. The participants who were receiving psychotropic medication (n = 8) were compared with the drug-free participants (n = 7). Given the small sample size, medication status was not included as a covariate in multivariable models to avoid overfitting. The overall direction and pattern of changes in hostile attribution bias, QOL, and salivary oxytocin reactivity were broadly similar between the medicated and drug-free participants, and no consistent pattern emerged to suggest a systematic medication-related effect on the main outcomes. Regarding comorbidities, subgroup analyzes were not performed because only two participants did not have a comorbid diagnosis, resulting in a highly unbalanced grouping that would yield statistically unreliable and difficult-to-interpret estimates.

### 3.2 Correlation analyzes between psychological and physiological indices

The correlation matrix is presented in **Table 4**. A positive correlation trend was observed between the rate of change in subjective QOL and the rate of change in hostile attribution bias scores (Spearman’s ρ = 0.597, p = 0.019). This suggests that participants whose hostile attribution bias tended not to improve or even worsened may have reported higher subjective satisfaction.

**Table 4:** Correlation analyzes of psychological change rates.

Variable		AIHQ_Hostile attribution bias	AIHQ_Aggressive response bias	AIHQ_Blame score	SRS-2_SCI	SRS-2_RRB	Subjective QOL
AIHQ_Hostile attribution bias	ρ	—					
	df	—					
	*p-value*	—					
AIHQ_Aggressive response bias	ρ	0.243	—				
	df	13	—				
	*p-value*	0.383	—				
AIHQ_Blame score	ρ	-0.198	0.304	—			
	df	13	13	—			
	*p-value*	0.478	0.271	—			
SRS-2_SCI	ρ	0.255	-0.015	-0.147	—		
	df	12	12	12	—		
	*p-value*	0.379	0.964	0.616	—		
SRS-2_RRB	ρ	-0.217	-0.06	0.13	0.219	—	
	df	12	12	12	12	—	
	*p-value*	0.457	0.84	0.657	0.453	—	
Subjective QOL	ρ	0.597*	0.325	-0.429	-0.248	-0.448	—
	df	13	13	13	12	12	—
	*p-value*	0.019	0.237	0.113	0.391	0.108	—

ρ:Spearman’s rank correlation coefficient, df:defrees of freedom, *p <.05, AIHQ = Ambiguous Intentions Hostility Questionnaire; SRS-2_SCI = Social Responsiveness Scale, Second Edition – Social Communication and Interaction subscale; SRS-2_RRB = Social Responsiveness Scale, Second Edition – Restricted and Repetitive Behavior subscale; QOL = Quality of Life.

## 4 Discussion

In this study, we examined the multidimensional psychological changes associated with a group CBT program that targets adolescents and young adults with ASD traits. The psychological indices included hostile attribution bias, subjective QOL, and social functioning (SRS-2).

The findings that group CBT led to significant improvements in hostile attribution bias, subjective QOL, and the SRS-2 SCI subscale scores suggest that group CBT can positively affect social cognition, life satisfaction, and interpersonal functioning. These results are consistent with those of previous studies (15, 16), supporting the applicability of group CBT, even for adolescents with ASD traits.

A notable contribution of the present study is that improvements were observed across multiple domains that are often only partially coupled: cognitive bias in ambiguous social situations (hostile attribution bias), subjective well-being (QOL), and caregiver-rated social functioning (SRS-2). Hostile attribution bias is theorized to amplify interpersonal threat perception and defensive responding, which may perpetuate conflicts and reduce opportunities for positive social learning. From this perspective, reducing hostile interpretations may create a more permissive cognitive context for engagement, perspective taking, and flexible problem solving, thereby supporting broader psychosocial gains. The observed improvement in SRS-2 Social Communication and Interaction (SCI) is consistent with this possibility and suggests that changes may extend beyond self-reported appraisals to caregiver-observed behavior in daily life.

However, an unexpected correlation emerged between the changes in hostile attribution bias and improvements in subjective QOL. Specifically, the participants who had smaller improvements, or even worsened, in hostile attribution bias tended to have greater improvements in subjective QOL (ρ = 0.60, *p* = .019). Although group CBT appeared to improve both hostile attribution bias and subjective QOL overall, the degree of change in hostile attribution bias itself may have negatively influenced subjective QOL. In other words, while group CBT reduced hostile interpretations, participants who engaged more intensely in self-reflection and made efforts to correct their biases may have experienced somewhat diminished improvements in their subjective QOL.

Several non-mutually exclusive interpretations may explain this discordant association. First, participation in CBT—particularly when it emphasizes identifying and correcting biased interpretations—may increase self-monitoring and metacognitive awareness. For some individuals, greater engagement in self-reflection could lead to heightened recognition of one’s own hostile interpretations and interpersonal difficulties, which may temporarily limit the apparent degree of improvement on hostile attribution measures even while perceived well-being improves through other aspects of the program (reassurance, normalization, acquisition of coping strategies, or improved communication). A previous study reported that the acquisition of adaptive skills and increased self-insight may not immediately translate into improvements in subjective well-being during the early phases of an intervention (21). Second, subjective QOL may have improved primarily via domains that are only indirectly captured by hostile attribution bias (increased self-efficacy, perceived support, reduced loneliness, or improved family/school relationships), resulting in QOL gains that are not contingent on large measurable shifts in hostile attribution bias. Third, QOL improvements may reflect changes in appraisal standards or priorities (response shift) during or after structured intervention, such that participants evaluate their well-being differently after gaining a new framework for understanding their experiences. Finally, because the present analyzes relied on change rates, baseline levels and regression-to-the-mean effects may have influenced individual change patterns; future studies should examine whether the association persists after accounting for baseline severity and using alternative longitudinal modeling approaches.

The group format itself may also be relevant for understanding why improvements in subjective QOL can occur even when reductions in hostile attribution bias are modest. Group CBT provides repeated opportunities for social learning in a structured, predictable environment, including peer modeling, feedback, validation, and shared problem solving. These group-specific elements may enhance perceived belongingness and interpersonal safety, which can improve subjective well-being even before cognitive bias shifts are fully consolidated or captured by questionnaire-based indices. In addition, group participation may facilitate behavioral changes (trying new interpersonal strategies, practicing skills) that translate into improved daily experiences and satisfaction, thereby increasing QOL independently of the degree of measurable change in hostile attribution bias.

Taken together, our findings suggest that group CBT targeting hostile attribution bias may be associated with improvements in hostile attribution bias, subjective QOL, and social communication in adolescents and young adults with ASD traits. At the same time, the relationship between cognitive-bias change and perceived well-being appears to be more complex than a simple causal chain, and QOL gains may emerge through multiple, partly distinct processes within CBT—potentially including group-specific mechanisms such as normalization, social support, and peer learning. Future studies with larger samples and controlled designs should test the replicability of the observed association between hostile attribution bias change and QOL change, and should clarify which components of group CBT are most strongly linked to improvements in social functioning and subjective well-being.

As an exploratory ancillary component, salivary oxytocin findings are provided in the **Supplementary Material** and were not used for mechanistic interpretation.

This study had a few limitations. First, the analyzes were conducted with 15 of the 21 participants because they completed all sessions. Restricting the analyzes to those who completed the sessions may have introduced bias by overestimating intervention effects. However, significant differences were not found between the completers (n = 15) and dropouts (n = 5) in demographic characteristics (age, sex) or pre-intervention psychological measures. Notably, although not significant, hostile attribution bias tended to be higher among the completers compared to dropouts (dropouts: 4.53 ± 2.36; completers: 5.79 ± 0.90; *p* = 0.086). Therefore, although attrition bias cannot be completely excluded, its impact on the interpretation of the results is likely limited. In addition, the small sample size (n = 15) restricted the statistical power, requiring cautious interpretation regarding the generalizability of the observed effects and trends. Second, the absence of a control group limited the ability to distinguish treatment effects from natural courses or external influences. Third, the small sample size limited our ability to formally evaluate the effects of comorbidities and concurrent medications using multivariable statistical models. Although exploratory subgroup analyzes for medication status did not indicate substantial differences in the overall pattern of the results, these analyzes were limited in statistical power and yielded heterogeneous findings. In addition, the highly unbalanced distribution of comorbidities precluded meaningful subgroup analyzes.

## 5 Conclusion

This pilot study demonstrated that group CBT focused on hostile attribution bias was associated with improvements in subjective QOL, objective social functioning, and hostile attribution bias in adolescents and young adults with ASD traits. Notably, although these outcomes improved concurrently at the group level, individual differences indicated that the magnitude of improvement in hostile attribution bias was not simply proportional to gains in subjective QOL, underscoring that changes in cognitive bias and perceived well-being may be linked through non-linear or partly distinct pathways. The group format may have contributed to these multidimensional improvements by providing structured opportunities for social learning, normalization, and peer support.

## Data Availability

The datasets presented in this study can be found in online repositories. The anonymized dataset generated and analyzed for this study can be found in Zenodo at https://doi.org/10.5281/zenodo.17213041.
