# Supplementary material for: Group CBT targeting hostile attribution bias in adolescents and young adults with ASD traits
Source: Front Psychiatry. 2026 Apr 22;17:1796850. doi: 10.3389/fpsyt.2026.1796850 (PMC13143942; doi:10.3389/fpsyt.2026.1796850)
Supplement: Supplementary file 1 [file Table1.docx]

**Supplementary Material**

**Salivary Oxytocin Analyses (Exploratory)**

This document accompanies the main manuscript reporting outcomes of an 8-session group cognitive behavioral therapy (CBT) program targeting hostile attribution bias and suspiciousness in adolescents and young adults with autism spectrum disorder (ASD) traits. Salivary oxytocin analyses are provided for completeness and continuity with the original submission history, but are presented strictly as exploratory findings and are not used to support mechanistic claims.

Given ongoing methodological debates regarding salivary oxytocin measurement—especially when assays are performed without solid-phase extraction—and the limited availability of independent validation data for non-extracted saliva matrices, these results should be interpreted with substantial caution. Archived saliva samples were not available for re-assay with extraction; therefore, concordance between extracted and non-extracted measurements could not be assessed in the current dataset.

# Supplementary Methods

## Salivary oxytocin sampling

Oxytocin was assessed using saliva samples rather than plasma samples for ethical and practical reasons. Saliva sampling is non-invasive and can be performed repeatedly with minimal burden, which is particularly important when working with adolescents with ASD traits who may have heightened sensitivity to medical procedures such as venipuncture. Peripheral oxytocin levels measured in different biological matrices are not interchangeable, and a single baseline measurement in either saliva or plasma is unlikely to provide a reliable trait marker of oxytocinergic function. Accordingly, the present analyses focused on within-person change rates around CBT sessions as an index of peripheral neuroendocrine reactivity.

Saliva samples were collected at four time points: before (16:00–16:30) and after (17:00–17:30) sessions 1 and 8 of the CBT program. Participants were screened to confirm that they had not eaten within 1 hour prior to sampling and had not received any dental treatment within the preceding 24 hours. Prior to collection, they rinsed their mouths with water to remove food residue and remained seated quietly for 10 minutes. Room temperature during saliva collection and CBT sessions was maintained at approximately 25 °C. Immediately after collection, aprotinin (100 KIU/mL; protease inhibitor) and ProClin 950 (1/750 v/v; preservative) were added to samples, which were then stored at −80 °C.

## Salivary oxytocin assay

Salivary oxytocin concentrations were measured using the Oxytocin ELISA Kit (FUJIFILM Wako Pure Chemical Corporation, Osaka, Japan), following the manufacturer’s protocol, and read using a SpectraMax i3 microplate reader (Molecular Devices, San Jose, CA, USA). All measurements were completed within 6 months of saliva collection.

The Wako Oxytocin ELISA Kit simplifies pretreatment and omits solid-phase extraction. While the manufacturer reports favorable analytic recovery in several matrices, independent peer-reviewed validation in saliva remains limited with respect to (i) agreement between extracted and non-extracted samples, (ii) parallelism in salivary matrix, and (iii) specificity for intact oxytocin versus potential interfering substances. Accordingly, oxytocin results are treated as exploratory and are presented in this Supplementary Material to preserve transparency about what was measured, without asserting biological mechanisms.

## Derived indices and missing data

For each CBT session (sessions 1 and 8), an oxytocin change rate (%) was calculated as (post-session value − pre-session value) / pre-session value × 100. The session 1 change rate was treated as a ‘pre-CBT’ index and the session 8 change rate as a ‘post-CBT’ index. The difference between the two change rates was defined as Δ oxytocin change rate, representing the change in oxytocin reactivity across the program.

In total, 60 saliva samples (15 participants × four time points) were collected. One sample obtained before session 8 could not be assayed successfully and was treated as missing. Pairwise deletion was applied for analyses involving oxytocin; consequently, the session 8 change rate and Δ oxytocin change rate could not be calculated for that participant.

# Supplementary Results

Summary statistics and inferential results for oxytocin indices are reported below. As in the main manuscript, psychological indices were analyzed using change rates; oxytocin indices are reported as within-session change rates and their difference across the program. All oxytocin-related analyses should be regarded as exploratory.

## Table S1. Salivary oxytocin change rates at sessions 1 and 8

| Measure | Session 1 change rate (pre-CBT) Median [Q1, Q3] | Session 8 change rate (post-CBT) Median [Q1, Q3] | p-value | Effect size (r) |
| --- | --- | --- | --- | --- |
| Oxytocin change rate (%) | -33.26 [-73.85, 25.31] | 13.77 [-16.76, 66.54] | 0.583 | 0.181 |

*Notes: Oxytocin change rates were derived as (post-session value − pre-session value) / pre-session value × 100. Because change rates were non-normally distributed, the Wilcoxon signed-rank test was used; effect size is rank-biserial correlation (r).*

## Table S2. Correlation matrix including Δ oxytocin change rate

|  | Δ oxytocin change rate | AIHQ_Hostile attribution bias | AIHQ_Aggressive response bias | AIHQ_Blame score | SRS-2_SCI | SRS-2_RRB | Subjective QOL |
| --- | --- | --- | --- | --- | --- | --- | --- |
| Δ oxytocin change rate | — |  |  |  |  |  |  |
| AIHQ_Hostile attribution bias | 0.414 | — |  |  |  |  |  |
| AIHQ_Aggressive response bias | 0.266 | 0.243 | — |  |  |  |  |
| AIHQ_Blame score | 0.191 | -0.198 | 0.304 | — |  |  |  |
| SRS-2_SCI | 0.527† | 0.255 | -0.015 | -0.147 | — |  |  |
| SRS-2_RRB | -0.055 | -0.217 | -0.06 | 0.13 | 0.219 | — |  |
| Subjective QOL | 0.108 | 0.597* | 0.325 | -0.429 | -0.248 | -0.448 | — |

*Spearman’s rank correlation coefficients (ρ) are shown. Symbols: † p < 0.10; * p < 0.05. Pairwise deletion was used; degrees of freedom vary across pairs due to missing oxytocin data at session 8 for one participant.*

## Table S3. Multiple regression predicting subjective QOL change (exploratory)

| Predictor | β | SE β | t | p-value |
| --- | --- | --- | --- | --- |
| Intercept | 0.203 | 0.242 | 0.840 | 0.420 |
| Hostile attribution bias change rate | 1.043 | 0.341 | 3.063 | 0.012 |
| Δ oxytocin change rate | 0.499 | 0.312 | 1.600 | 0.141 |
| Hostile attribution bias × Δ oxytocin | -1.908 | 0.980 | -1.948 | 0.080 |

*Model summary: R² = 0.501; adjusted R² = 0.352; F(3,10) = 3.353; p = 0.064; n = 14. All variables were standardized (z-scores) prior to analysis; β indicates standardized coefficients. Interpret as exploratory due to limited sample size and measurement constraints.*

## Table S4. Multiple regression predicting SRS-2 SCI change (exploratory)

| Predictor | β | SE β | t | p-value |
| --- | --- | --- | --- | --- |
| Intercept | -0.711 | 0.449 | -1.582 | 0.148 |
| Hostile attribution bias change rate | 0.046 | 0.473 | 0.098 | 0.924 |
| Δ oxytocin change rate | 3.054 | 2.545 | 1.200 | 0.261 |
| Hostile attribution bias × Δ oxytocin | -0.530 | 2.396 | -0.221 | 0.830 |

*Model summary: R² = 0.508; adjusted R² = 0.343; F(3,9) = 3.092; p = 0.082; n = 13. All variables were standardized (z-scores) prior to analysis. Multicollinearity was high in the original model including the interaction term (maximum VIF ≈ 16.9); results should be interpreted with particular caution.*

# Supplementary Discussion

In these exploratory analyses, oxytocin change rates did not differ significantly between the first and final CBT sessions, and associations between oxytocin indices and psychological outcomes were not statistically significant at the bivariate level. These findings may reflect true null associations, limited statistical power (n = 13–14 for regression models), and/or measurement noise in peripheral oxytocin assays.

Prior studies of psychosocial interventions and oxytocin have reported heterogeneous responses (increases, decreases, or no changes), suggesting that peripheral oxytocin may be sensitive to contextual factors such as novelty, social arousal, and stress regulation. Group-based interventions, in particular, may evoke endocrine responses related to interpersonal context rather than directly indexing symptom change. Therefore, even when within-person change rates are quantified, oxytocin measures alone may be insufficient to explain therapeutic effects in complex psychosocial interventions.

Interpretation is further constrained by methodological considerations in salivary oxytocin measurement. Without solid-phase extraction and independent validation in saliva, it remains uncertain whether measured values reflect intact oxytocin or non-specific immunoreactivity influenced by the complex salivary matrix. Because archived samples were not available, we could not perform extraction-based re-assay or evaluate agreement between extracted and non-extracted measurements in this study. Accordingly, these results should be viewed as preliminary descriptive data that may inform future studies employing validated protocols, larger samples, and pre-specified mechanistic hypotheses.

# Supplementary References (Oxytocin-related)

McCullough ME, Churchland PS, Mendez AJ. Problems with measuring peripheral oxytocin: can the data on oxytocin and human behavior be trusted? Neurosci Biobehav Rev. 2013;37(8):1485–1492.

Horvat-Gordon M, Granger DA, Schwartz EB, Nelson VJ, Kivlighan KT. Oxytocin is not a valid biomarker when measured in saliva by immunoassay. Physiol Behav. 2005;84(3):445–448.

Leng G, Sabatier N. Measuring oxytocin and vasopressin: bioassays, immunoassays and random numbers. J Neuroendocrinol. 2016;28(10).

Brandtzaeg OK, Johnsen E, Roberg-Larsen H, et al. Proteomics tools reveal startlingly high amounts of oxytocin in plasma and serum. Sci Rep. 2016;6:31693.

Tabak BA, Leng G, Szeto A, et al. Advances in human oxytocin measurement: challenges and proposed solutions. Mol Psychiatry. 2023;28:127–140.

FUJIFILM Wako Pure Chemical Corporation. Oxytocin ELISA Kit Instruction Manual. 2022.

Shamay-Tsoory SG, Abu-Akel A. The Social Salience Hypothesis of Oxytocin. Biol Psychiatry. 2016;79:194–202.

Hohl CH, Zilcha-Mano S, Delgadillo J. Is the “social hormone” oxytocin relevant to psychotherapy treatment outcomes? A systematic review of observational and experimental studies. Neurosci Biobehav Rev. 2024;167:105935.

Zierhut M, Bergmann N, Hahne I, et al. The combination of oxytocin and mindfulness-based group therapy for empathy and negative symptoms in schizophrenia spectrum disorders—a double-blinded, randomized, placebo-controlled pilot study. J Psychiatr Res. 2024;171:222–229.

Böge K, Bergmann N, Zierhut M, et al. The relationship between mindfulness and empathy with the oxytocinergic system in persons with schizophrenia spectrum disorders—the OXYGEN trial. Int J Clin Health Psychol. 2024;24:100503.

Bellosta-Batalla M, Blanco-Gandía MC, Rodríguez-Arias M, et al. Brief mindfulness session improves mood and increases salivary oxytocin in psychology students. Stress Health. 2020;36:469–477.

Schladt TM, Nordmann GC, Emilius R, et al. Choir versus solo singing: effects on mood, and salivary oxytocin and cortisol concentrations. Front Hum Neurosci. 2017;11:430.

Geva N, Uzefovsky F, Levy-Tzedek S. Touching the social robot PARO reduces pain perception and salivary oxytocin levels. Sci Rep. 2020;10:9814.

Qian M, Wang M, Song S, et al. Investigating the psychophysiological effects of NaiKan Therapy: salivary oxytocin and cortisol release. Front Integr Neurosci. 2025;19:1476654.
